# Application of Mivacurium in Fast-Track Anesthesia for Transthoracic Device Closure of Ventricular Septal Defects in Children

**DOI:** 10.21470/1678-9741-2020-0580

**Published:** 2022

**Authors:** Jing Wang, Yu-Qing Lei, Jian-Feng Liu, Zeng-Chun Wang, Hua Cao, Qiang Chen

**Affiliations:** 1 Department of Cardiac Surgery, Fujian Maternity and Child Health Hospital, Affiliated Hospital of Fujian Medical University, Fuzhou, People’s Republic of China.; 2 Fujian Key Laboratory of Women and Children’s Critical Diseases Research, Fujian Maternity and Child Health Hospital, Fuzhou, People’s Republic of China.; 3 Fujian Branch of Shanghai Children’s Medical Center, Fuzhou, People’s Republic of China.; 4 Fujian Children’s Hospital, Fuzhou, People’s Republic of China.; 5 Department of Cardiovascular Surgery, Union Hospital, Fujian Medical University, Fuzhou, People’s Republic of China.

**Keywords:** Anesthesia, Cardiac Procedures, Ventricular Heart Septal Defects, Transthoracic Device, Children, Mivacurium, Atracurium, Child

## Abstract

**Introduction:**

The objective of this study was to investigate the effect of mivacurium in the application of fast-track anesthesia for transthoracic device closure of ventricular septal defects (VSDs) in children.

**Methods:**

The data of 108 children who underwent transthoracic device closure of VSDs from December 2018 to June 2020 were recorded and analyzed. All children were divided into group M (mivacurium group, n=55) and group C (cisatracurium group, n=53) according to the different muscle relaxant drug used.

**Results:**

No statistically significant differences in general preoperative data, intraoperative hemodynamic changes, or the incidence of adverse reactions were noted between the two groups (*P*>0.05). However, the intubation condition rating of children in group M was better than that in group C. The onset time, duration of clinical action and recovery index of the muscle relaxant, postoperative mechanical ventilation duration, and length of intensive care unit stay in group M were significantly lower than those in group C (*P*<0.05).

**Conclusion:**

It is safe and feasible to use mivacurium as a muscle relaxant in children undergoing fast-track cardiac anesthesia during transthoracic device closure of VSDs.

**Table:** 

Abbreviations, acronyms & symbols	
HR	= Heart rate
ICU	= Intensive care unit
MAP	= Mean arterial pressure
TEE	= Transesophageal echocardiography
VSD	= Ventricular septal defects

## INTRODUCTION

Ventricular septal defect (VSD) is one of the most common congenital heart diseases^[1]^. Commonly used treatments include surgical repair under cardiopulmonary bypass and transcatheter device closure guided by echocardiography and fluoroscopy. In recent years, transthoracic device closure of VSD has been widely used in the treatment of VSD and accepted by children and their families given its small incision, lack of a need for cardiopulmonary bypass, lack of radiation exposure, short operation time, fast postoperative recovery, and few complications^[2,3]^. Transthoracic device closure of VSD guided by transesophageal echocardiography (TEE) combined with fast-track cardiac anesthesia technology may promote the rapid postoperative recovery of patients^[4,5]^. Fast-track cardiac anesthesia involves selecting the appropriate anesthesia methods and drugs to allow patients to undergo extubation immediately or as soon as possible (within six hours after the operation) to reduce the length of the postoperative intensive care unit (ICU) stay, hospitalization stay, and total medical costs^[6]^. Many studies on fast-track cardiac anesthesia have focused on the use of opioids but ignored the choice of muscle relaxants. The reasonable choice of muscle relaxants is another key to the success and safety of fast-track anesthesia for children. As a short-acting non-depolarizing muscle relaxant, mivacurium has a short action time, rapid recovery, minimal accumulation in the body, and minimal adverse reactions in the nervous system and cardiovascular system[^7,8]^]. This study aimed to investigate the effect of mivacurium in fast-track cardiac anesthesia for transthoracic device closure of VSDs in children.

## METHODS

The present study was approved by the ethics committee of our hospital (2020KY039) and adhered to the Declaration of Helsinki. Besides, written informed consent was obtained from the patient’s parents.

This is a retrospective study conducted in two teaching hospitals. Between December 2018 and June 2020, 108 children who underwent transthoracic device closure of VSD through a small chest incision were selected as the research object. The inclusion criteria were as follows: 1) simple perimembranous VSD and suitable for undergoing device closure; 2) cisatracurium or mivacurium was used as an inducer of muscle relaxants during the operation; 3) no pulmonary infection or abnormal pulmonary function before the operation. The exclusion criteria were: 1) VSD combined with other cardiac malformations that require surgical correction; 2) failure of transthoracic device closure of VSD and transfer to surgical repair; 3) children with severe pulmonary hypertension.

All the enrolled children completed routine preoperative examinations. Family members were informed of the advantages, disadvantages, and indications of the two anesthesia regimens the day before the surgery. Then, according to the patient’s condition and the family’s wishes, the surgeon and the anesthesiologist jointly chose the patient’s anesthesia plan. The patients were divided into two groups according to the different muscle relaxants used in operation. In total, 55 children were included in group M (mivacurium), and 53 children were included in group C (cisatracurium) (Table 1).

**Table 1:** Demographic and clinical characteristics of the two groups.

	Group M	Group C	*P*-value
Number of patients	55	53	
Gender (male/female)	25/30	24/29	0.50
Age (years)	2.6±1.0	2.8±1.0	0.54
Weight (kg)	18.6±2.9	19.1±2.5	0.33
Operation time (minutes)	49.9±10.6	50.3±7.0	0.41

All children fasted for 4~6 hours and did not consume water for 2~4 hours before the anesthesia. The children were administered oral midazolam syrup (0.5 mg/kg) 30 minutes before entering the operating room. After entry into the operating room, the venous channel was established, and physiological saline was infused. Heart rate (HR), noninvasive blood pressure, and oxygen saturation were monitored. Midazolam (0.05 mg/kg) and fentanyl (10 µg/kg) were intravenously injected for anesthesia induction. After the patient lost consciousness, a Veryark-TOF neuromuscular monitor was turned on to monitor the thumb. A baseline record of neuromuscular monitoring was generated. Neuromuscular transmission was monitored with acceleromyograph. The ulnar nerve was stimulated at the wrist by a train-of-four stimulation (current: 50 mA, duration: 0.2 ms, frequency: 2 Hz, interval between strings: 15 seconds), and neuromuscular function was measured at the adductor pollicis. The acceleration indicator was fixed to the palm side of the thumb’s non-infusion side, and the skin surface electrode was placed on the ulnar side of the left forearm, near the wrist. After the first twitch was stabilized at 100%, mivacurium (0.2 mg/kg) was intravenously injected in group M with an injection speed > 30 seconds, and cisatracurium (0.15 mg/kg) was intravenously injected in group C. When the first twitch inhibition of train-of-four reached 95%, tracheal intubation was performed. After successful intubation and mechanical ventilation, radial artery and subclavian vein catheterization were performed. The invasive mean arterial pressure (MAP), central venous pressure, end-expiratory carbon dioxide, nasopharyngeal temperature, and arterial blood gas analysis were monitored during the procedure. Anesthesia was maintained by intravenous pump infusion of 0.3-0.5 ug/kg/min remifentanil and 2-3% sevoflurane via inhalation. When the first twitch recovered to 25%, the children in the two groups were injected with corresponding muscle relaxant at a dose of 0.1 mg/kg to maintain muscle relaxation, and the sedative dose was adjusted according to the depth of anesthesia during the operation.

During the procedure, a small incision was made at the lower portion of the sternum. The pericardium was cut open to expose the free wall of the right ventricle, and 1 mg/kg heparin was administered. The puncture site was located at the free wall of the right ventricle under TEE guidance. Then, a purse-string suture was placed at this site. A delivery pathway through the right ventricle-VSD-left ventricle was established under TEE guidance. An occluder was released through the delivery pathway to close the VSD. Then, TEE was used to confirm the location of the occluder and the cardiac function. After the operation, the patient was sent to the ICU for further monitoring and treatment.

Hemodynamic indexes, including HR and blood pressure, were recorded at five time points — before anesthesia induction (T1), during tracheal intubation (T2), during skin incision (T3), during thoracotomy (T4), and during incision closing (T5) — in both groups (Table 2). The tracheal intubation conditions of both groups were also recorded, and the intubation conditions were evaluated based on the Krieg scale, an endotracheal intubation rating method: level 1, excellent, with loose vocal cords; level 2, good, vocal cords relaxed, mild cough from the endotracheal tube; level 3, poor, moderate adduction of vocal cords, significant cough when trachea passes; and level 4, unable to complete the intubation[^9]^]. The related results were shown in Table 3. The neuromuscular effects were recorded: (1) onset time, time from the first injection of muscle relaxant to the maximum inhibition of the first twitch; (2) duration of clinical action, time from muscle relaxant administration to 25% recovery of the first twitch; and (3) recovery index, the recovery time of the first twitch from 25 to 75%. The related results were shown in Table 3. Adverse reactions, such as bronchospasm and skin flushing, postoperative mechanical ventilation duration, and length of ICU unit stay were recorded in both groups and presented in Table 3.

**Table 2:** Comparison of perioperative hemodynamics of the two groups.

	Group M	Group C	*P*-value
T1			
MAP (mmHg)	54.4±3.8	53.6±4.1	0.31
HR (bpm)	116.2±8.5	118.2±7.5	0.19
T2			
MAP (mmHg)	49.8±3.3	50.0±3.3	0.72
HR (bpm)	110.3±7.9	111.7±7.6	0.35
T3			
MAP (mmHg)	57.0±4.9	56.7±3.4	0.73
HR (bpm)	123.3±9.8	124.8±9.4	0.43
T4			
MAP (mmHg)	57.9±4.0	59.0±3.4	0.14
HR (bpm)	117.4±7.6	116.2±6.3	0.41
T5			
MAP (mmHg)	55.0±3.1	55.9±3.9	0.16
HR (bpm)	109.8±9.0	111.7±9.0	0.22

HR=heart rate; MAP=mean arterial pressure T1=before anesthesia induction; T2=during endotracheal intubation; T3=during skin incision; T4=during thoracotomy; T5=closing the incision

**Table 3:** Comparison of intubation conditions, effect of muscle relaxation, and postoperative data and adverse reactions of the two groups.

Parameter	Group M	Group C	*P*-value
Number of patients	55	53	
Level 1: case (%)	53 (96.4%)	39 (73.6%)	0.03
Level 2: case (%)	2 (3.6%)	9 (17.0%)	
Level 3: case (%)	0 (0%)	5 (9.4%)	
Onset time (seconds)	157.6±26.8	192.3±28.8	*P*<0.001
Duration of clinical action (minutes)	13.7±3.2	34.4±4.8	*P*<0.001
Recovery index (minutes)	5.4±1.5	12.2±3.4	*P*<0.001
Mechanical ventilation duration (hours)	0.6±0.2	1.6±0.7	*P*<0.001
Length of ICU stay (hours)	3.7±1.3	5.7±2.4	*P*<0.001
Bronchospasm: case (%)	0 (0)	0 (0)	-
Erubescence: case (%)	0 (0)	0 (0)	-

ICU=intensive care unit

### Statistical Analysis

The continuous data exhibited normal distribution as assessed by the normality test and were statistically analyzed using an independent sample *t*-test. The Chi-square test was used to compare the number of postoperative complications between the two groups. A *P*-value < 0.05 was defined as statistically significant.

## RESULTS

No statistically significant differences in age, gender, body weight, and operation time were noted between the two groups (*P*>0.05) (Table 1). As shown in Table 2, the hemodynamic indexes of the two groups before anesthesia induction (T1), tracheal intubation (T2), skin incision (T3), thoracotomy (T4), and close the incision (T5) were stable and not statistically significant (*P*>0.05). As shown in Table 3, the intubation condition rating of children in group M was better than of those in group C, and the difference was statistically significant (*P*<0.05). There was no statistically significant difference in the incidence of adverse reactions (*P*>0.05). Bronchospasm and skin flushing did not occur in either group. The onset time, duration of clinical action, recovery index, postoperative mechanical ventilation duration, and length of ICU stay in group M were significantly shorter than those in group C (*P*<0.05).

## DISCUSSION

VSDs is one of most common congenital heart diseases^[10]^. The traditional treatment of VSD is surgical repair under cardiopulmonary bypass. However, such operation might be associated with systemic inflammatory reactions, sizeable surgical incision, and potential damage to other organs^[11]^.Recently, transthoracic device closure of VSD guided by TEE has gained popularity in China, and the procedure is completed through a small incision in the lower sternum and uses a specially designed delivering sheath. Besides, intraoperative TEE is used to assess whether a residual shunt was present or a change in cardiac morphology was caused by the occluder. This procedure’s advantages were as follows: no cardiopulmonary bypass, small surgical incision, short operation time, quick postoperative recovery, and easy to learn. Thus, fast-track cardiac anesthesia is implemented by anesthesiologists to support these surgical techniques, to extubate these patients as soon as possible after surgery, to reduce the length of ICU stay, and to promote rapid postoperative recovery.

The concept of fast-track anesthesia for cardiac surgery was proposed in the 1990s and has been continuously improved and perfected in recent years. Its safety and effectiveness in pediatric cardiac surgery had been confirmed in many studies^[12,13]^. Fast-track cardiac anesthesia is employed to achieve early postoperative extubation, reduce complications by optimizing anesthetic drugs and applying various techniques, and meet the optimal anesthesia effect required for cardiac surgery. Iezzi et al. found that early extubation (four hours) in pediatric congenital heart disease was safe and effective because it reduced the time of intubation and ventilator use and did not increase postoperative complications^[14]^. In the early stage, large doses of opioids were mostly used to achieve the anesthetic effect required by cardiac surgery. In recent years, clinical studies on fast-track cardiac anesthesia were mostly focused on optimizing the use of opioids, while less attention was paid to the application of muscle relaxants.

Mivacurium is a short-acting non-depolarizing muscle relaxant. After entering the blood circulation, plasma cholinesterase can be rapidly degraded, and the plasma clearance half-life is only 2.6 minutes. Due to the short duration of action, mivacurium has become the main muscle relaxant used for pediatric anesthesia in Germany^[8]^. The elimination of mivacurium does not directly depend on liver and kidney functions, and the decomposition products do not have muscle relaxation effects. Therefore, mivacurium has almost no accumulation effect in the body and has a slight effect on circulation^[15]^. The operation time of transthoracic device closure of VSD in children is relatively short, so the pharmacodynamic characteristics of mivacurium are consistent with such procedure’s requirements. Cisatracurium is a medium-short-acting muscle relaxant. Given its weak histamine release effect, minimal effect of conventional dosage on liver and kidney function, and minimal effect on circulation, it is also often used for anesthesia in cardiac surgery.

In this study, no significant differences in age, gender, body weight, and operation time were noted between the two groups, demonstrating that the two groups of data were comparable. The intubation condition rating in group M was better than that in group C, and the percentage of children with level 1 intubation condition reached 96.4%, suggesting that mivacurium might be more conducive to obtain good intubation conditions. The onset time, duration of clinical action, and recovery index of muscle relaxation in group M were significantly shorter than those in group C. Such results indicated that mivacurium had a faster effect, no accumulation, shorter duration of action, and faster recovery. This study also found that the length of postoperative mechanical ventilation and ICU stay were shorter in group M, suggesting that mivacurium was more beneficial to postoperative recovery.

Mivacurium exhibits a dose-dependent adverse effect of histamine release. A single large-dose injection and rapid administration of mivacurium can cause histamine release, resulting in capillary dilatation, skin flushing (face and trunk), decreasing blood pressure, and increasing HR, and inducing laryngeal spasms. Compared with adults, the histamine release is less effective and has less of an effect on children’s blood pressure and HR^[16]^. The release of histamine is related to injection dose and injection speed^[17]^. Studies had shown that slow intravenous administration of mivacurium within 30-60 seconds could suppress histamine in the blood and reduce skin flushing^[18]^. In this study, hemodynamic indicators, such as MAP and HR, were stable during and after anesthesia induction in the two groups. The bolus injection rate may be slower (> 30 seconds) when mivacurium is administered in this study, which inhibits histamine release, thereby reducing hemodynamic fluctuations. Goosens et al. believed that mivacurium’s use did not change the hemodynamics and left cardiac function of patients with coronary artery disease^[19]^. Given that the infusion speed was > 60 seconds, there was a protective effect on myocardial function. Therefore, we should pay attention to the infusion speed when using mivacurium.

### Limitations

This article has many shortcomings. First, this was a retrospective study with relatively small sample size. Some of the evaluation indicators used were also subjective, which might lead to the results’ deviation. Second, this was not a prospective randomized, double-blind controlled study. In fact, in the current clinical work in China, the doctor-patient relationship was quite tense, so it was challenging to implement a prospective randomized, double-blind controlled study. Although the statistical value of our results might be inferior to that of prospective studies, we still believed that our study had some guiding clinical value. We hope to complete a multicenter prospective randomized, double-blind controlled study to verify our conclusions further.

## CONCLUSION

It is safe and feasible to use mivacurium as a muscle relaxant in children to perform fast-track cardiac anesthesia during transthoracic device closure of VSD. The effect of mivacurium is slightly faster. Besides, the duration is shorter, and the recovery is faster. However, it should be noted that mivacurium injection should be slow, lasting at least 30 seconds.

**Table:** 

Authors' roles & responsibilities	
JW	Substantial contributions to the design of the work; drafting the work; final approval of the version to be published
YQL	Substantial contributions to the acquisition and analysis of data for the work; final approval of the version to be published
JFL	Substantial contributions to the acquisition and analysis of data for the work; final approval of the version to be published
ZCW	Substantial contributions to the acquisition and analysis of data for the work; final approval of the version to be published
HC	Revising the work; final approval of the version to be published
QC	Substantial contributions to the design of the work; final approval of the version to be published
